# Evidence for a diagnostic distinction between functional seizures and functional motor symptoms from the TriNetX electronic health record database

**DOI:** 10.1017/S0033291726103456

**Published:** 2026-02-26

**Authors:** Richard A. Kanaan, Timothy R. Nicholson, Livia Asan, Thomas A. Pollak

**Affiliations:** 1 Department of Psychiatry, University of Melbourne, Austin Hospital, Heidelberg, Australia; 2 Neuropsychiatry Research and Education Group, Institute of Psychiatry, Psychology and Neuroscience, London, United Kingdom; 3 Department of Neurology and Center for Translational Neuro- and Behavioral Sciences (C-TNBS), University Hospital Essen, Essen, Germany; 4 Department of Psychosis Studies, King’s College London, Institute of Psychiatry, Psychology and Neuroscience, London, United Kingdom

**Keywords:** etiology, functional neurological disorder, mechanism, nosology

## Abstract

**Background:**

There is evidence that the two most common subtypes of functional neurological disorder, functional seizures (FSs) and functional motor symptoms (FMDs), have differences between them beyond symptom type, creating debate as to whether they may best be considered distinct disorders. However, most research has studied FS or FMD separately, and the few studies that have directly compared them have been relatively small. We used the large TriNetX electronic health database to see whether the differences previously identified would be confirmed in a larger sample of both subtypes.

**Methods:**

All cases of FMD without FS were compared with cases of FS without FMD, extracted from the TriNetX electronic health records database. Previously identified between-group differences in demographics, comorbidity, and antecedents were compared between groups.

**Results:**

Over 120,000 cases of FMD and FS were extracted. They confirmed that people with FS were significantly younger and had a younger onset than those with FMD, were more likely to be Black and less likely to be Asian, and had higher rates of all comorbid mental health diagnoses, other than somatoform diagnoses, which were more common in FMD. The onset of FS was more commonly preceded by psychological injury, as measured by preceding depression or stress reactions.

**Conclusion:**

The differences between FMD and FS previously identified in small studies were confirmed in this much larger dataset. They provide indirect support for differences in etiology and mechanism, which may in turn support a nosological distinction between FMD and FS.

## Introduction

Functional neurological disorder (FND) presents with a variety of neurological symptoms, often with several in the same patient, often changing over time, and often accompanied by other physical and psychological symptoms not normally considered a core part of the disorder. Distinct symptom subtypes are described in the diagnostic manuals (APA, 2013; WHO, 2018), with functional seizures (FSs) or functional motor symptoms (FMDs) the most common (Ljungberg, 1957). Indeed, most FND research and clinical reporting typically focuses on one or the other of these subtypes in isolation. While this separation may simply reflect pragmatic division along service lines or a desire to homogenize research samples, significant differences have been observed between the two subtypes. This has led to an ongoing debate as to whether the two subtypes may be best considered separate disorders.

The division of pathology into distinct disorders has traditionally been the foundational step in medicine. Only when a disease is studied separately from other diseases can its specific etiology, mechanism, and treatments be identified. This raises the obvious problem of deciding what the optimum division is before any specifics are identified – a particular problem in psychiatry and other areas where the disease processes are either unknown or highly complex. It previously led to calls to treat all the functional syndromes as a single disorder because there was no disease mechanism to separate them, their symptoms overlapped extensively, their demographics were similar, and even their treatments were essentially the same (Wessely, Nimnuan, & Sharpe, 1999). The counterargument to this was that even subtle differences in symptoms, demographics, and etiology were actually clues to differences in mechanism, the standard by which division should ultimately be decided, and that treatments should be tailored accordingly (Kanaan, Lepine, & Wessely, 2007; Wessely & White, 2004).

The relationship between FS and FMD is a little different. While there is substantial symptom overlap between them, and their mechanisms are still the subject of speculation, there is already a substantial difference in how they are treated. FSs are typically treated with psychological therapy; FMDs are typically treated with physiotherapy. It is possible to explain these as practice differences arising from the distinct clinical traditions of the epilepsy and movement disorder clinics where FS and FMD tend to be assessed. Harder to explain are the reported demographic or etiological differences. We reviewed these in 2017 (Kanaan, Duncan, Goldstein, Jankovic, & Cavanna, 2017), and others have contributed comparative data since (Huepe-Artigas, Carter, Morsy, & Kanaan, 2020; Huepe-Artigas, Singh, Weinberg, & Kanaan, 2022; Lidstone et al., 2022; Ludwig et al., 2018; Mishra & Pandey, 2024; Morsy et al., 2022). These suggested that people with FS are younger, and present earlier (Kanaan et al., 2017; Lidstone et al., 2022); that people with FS may have higher rates of childhood abuse, particularly sexual abuse (Kanaan et al., 2017); that people with FS have higher rates of various mental disorders, such as borderline personality disorder, anxiety, dissociation, and suicidality (Huepe-Artigas et al., 2020; Kanaan et al., 2017); that the rates and types of events preceding onset may differ, with psychological stressors more common preceding FS, but medical events impacting the limbs, headaches, and other functional somatic syndromes more common preceding FMD (Huepe-Artigas et al., 2022; Ludwig et al., 2018; Mishra & Pandey, 2024; Morsy et al., 2022); and that the ratio of FS/FMD may be higher in non-western societies (Kanaan et al., 2017). Together, they suggest that FS and FMD occur in somewhat different people and have somewhat different etiologies. These would be more fundamental differences than mere differences in clinical practice, and as clues to potential differences in mechanism, would provide more substantial support for a separate classification.

However, the available data are still inadequate to support such an argument. As noted, the great majority of studies in FND consider either FMD or FS alone, preventing direct comparison. Studies drawn from one subtype are likely to involve distinct selection biases from those drawn from the other subtype, as the services will have distinct recruitment routes and barriers to entry. The studies that do include both subtypes are comparatively few and are all relatively small. Though they include three meta-analyses (Lidstone et al., 2022; Ludwig et al., 2018; Morsy et al., 2022), these are largely of small studies, and none include statistical comparisons between FMD and FS.

One possible solution to this lack of data would be the use of a large-scale clinical database, where patients are enrolled across entire health services rather than from clinical departments. One such database is TriNetX, a data repository including the electronic health records of over 150 million patients from 19 countries at the time of writing (http://trinetx.com). In this study, we present an interrogation of the TriNetX database, comparing FS and FMD on the differences previously reported, and others that the scale of the database allows, to see whether the distinction between FS and FMD can be sustained and extended. We hypothesized that FS and FMD would be demographically different, as would their early and recent antecedents, and current comorbidities, in line with previous reports: that people with FS will show younger age and onset; higher rates of psychiatric comorbidity; lower rates of functional somatic syndromes; higher rates of preceding psychological trauma, both recent and remote; lower rates of preceding medical events affecting the limbs; and will be relatively more common in non-western societies.

## Methods

### Study population

The TriNetX Research Network contains a set of delimited, de-identified electronic health records. The data available for analysis include demographic information and clinical codes for diagnoses, medications, and procedures, which are all time-indexed. The data are updated live, so that the total number of patients and health care organizations available varies over time, depending on which are online. We accessed the database on 2 April 2025, at ~1300 Australian Eastern Time: at that time, TrinextX was reporting data from 167,420,850 patients from 144 health care organizations. We searched for individuals with a diagnosis of FS but no diagnosis of FMD at any time using the standard international diagnostic coding system (International Classification of Diseases, Tenth Revision, Clinical Modification (ICD-10-CM) codes F44.5 and F44.4, respectively) and compared them with individuals with a diagnosis of FMD and no diagnosis of FS. This retrospective study is exempt from obtaining informed consent, and institutional ethical approval was not required according to national guidelines. The data analyzed are secondary data, do not involve intervention or interaction with human subjects, and have been de-identified in line with the de-identification standard outlined in Section §164.514(b)(1) of the HIPAA Privacy Rule (http://trinetx.com).

### Measures

We examined ICD-10-CM main categories (designated by letters) and selected subcategory root codes, particularly focusing on disorders within the mental and behavioral disorders (F category) and other somatic and pain root codes as suggested by previous studies. We considered the main surgical procedure categories using current procedural terminology codes. Codes made under ICD-9-CM are mapped to ICD-10-CM codes using the General Equivalence Mappings from the Centers for Medicare & Medicaid Services. Comorbid conditions were captured if diagnosed at any time up to and including the index diagnosis. Potential precipitants were considered any of the hypothesized diagnoses or procedures in the 6 months before the index diagnosis (including the day of diagnosis itself) compared with a year earlier (18–12 months before the index diagnosis). The 6-month window was chosen as a practical timeframe that would allow the capture of a reasonable number of events that might still be plausibly considered causal.

### Statistical analysis

We report the total numbers and proportions of patients from each cohort who had the corresponding diagnostic code(s). Comparisons between cohorts used chi-squared tests, independent samples *t*-tests, and *Z*-tests. After demographic characteristics were calculated, the cohorts were matched one-to-one on demographic variables using propensity scores from the specified covariates for subsequent analyses. Bonferroni correction was applied to each analysis. Analyses were conducted using the TrinetX statistical engine, with additional analyses in SPSS 30.0.0.0.

## Results

### Baseline characteristics

#### Demographics

**Figure 1.**
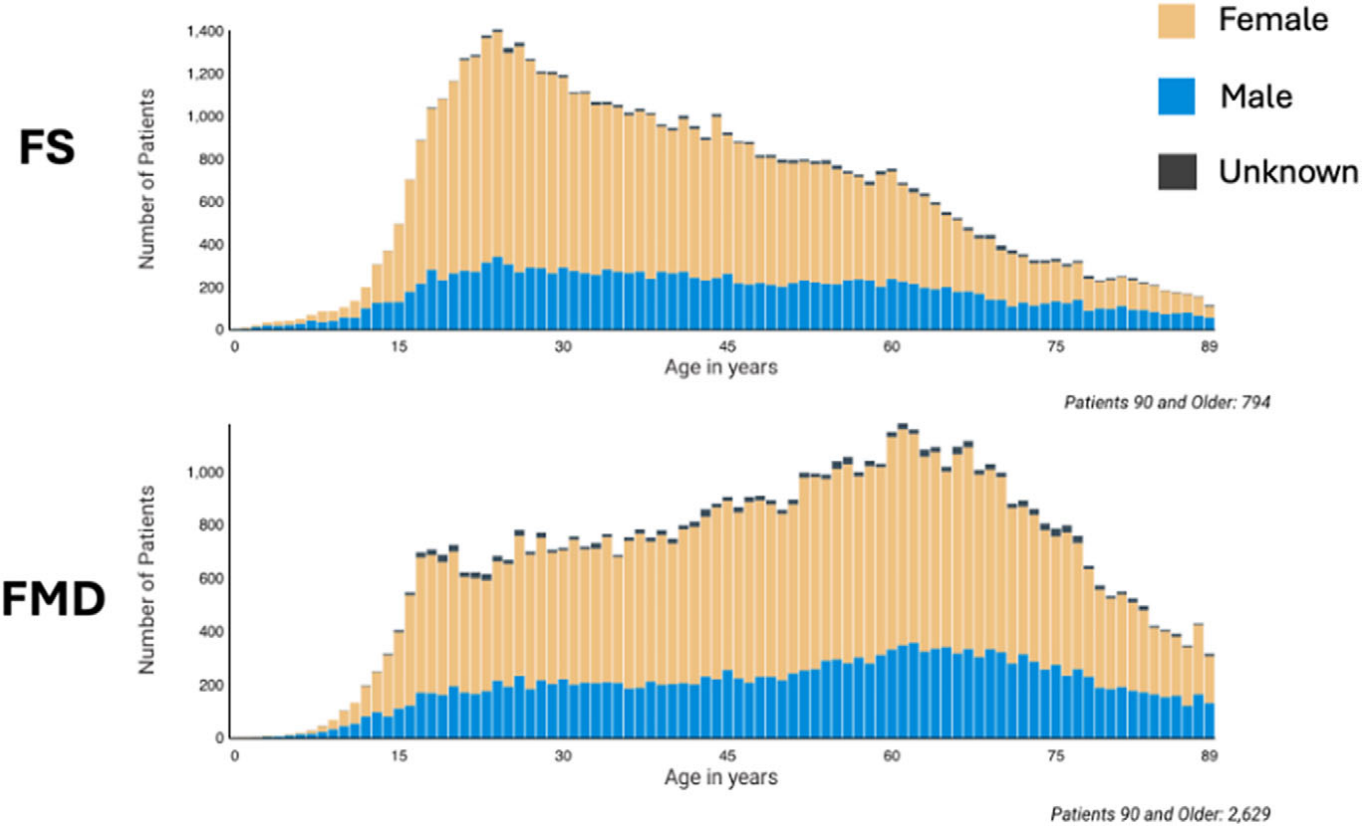
Age distribution of functional seizures (FSs) and functional motor symptoms (FMDs) divided by gender. Women are shown in the lighter color, men in the darker color, and those whose gender is unknown are shown in black.

At the time of access, the database contained 62,889 people with FMD without FS (~79% of the total number of patients with FMD), and 57,658 people with FS without FMD (~78% of the total number of patients with FS). We hereafter refer to these extracted samples as FMD and FS, respectively, for the sake of simplicity. Their demographics showed statistically significant but very small differences in race, ethnicity, and gender, and small-medium differences in current age (51 vs. 42 years) and age at diagnosis (44 vs. 37 years), with FMD being less White or Black, more Asian or Hispanic/Latino, and older, as shown in Table 1 and Figure 1. The age distributions differed more substantially in skew, with the modal age for FS 23 years and for FMD 61 years for both men and women. A sensitivity analysis, repeating the comparison without excluding those with both diagnoses, found similar, though smaller effects. Following this analysis, developmental factors and baseline comorbidity were calculated on subsets propensity matched for age at diagnosis, sex, race, and ethnicity (52,279 in each group).

**Table 1.** Demographics of functional motor symptoms (FMDs) and functional seizures (FSs)

Characteristic	FMD, *n* (%)	FS, *n* (%)	Std Diff	*p*-value
**Age (mean [SD])**	**51 (20.7)**	**42.5 (19.4)**	**0.4253**	**<0.0001** ^a^
**Age at diagnosis (mean [SD])**	**43.5 (20.6)**	**37.3 (19.2)**	**0.311**	**<0.0001** ^a^
Female	36,432 (69)	37,319 (71)	0.0441	<0.0001^a^
Male	15,785 (30)	14,488 (28)	0.0512	<0.0001^a^
Unknown gender	700 (1)	852 (2)	0.0245	<0.0001^a^
White	31,229 (59)	28,928 (55)	0.0825	<0.0001^a^
Black	7,890 (15)	9,501 (18)	0.0845	<0.0001^a^
Asian	2,628 (5)	790 (2)	0.1969	<0.0001^a^
Pacific Islander	310 (1)	635 (1)	0.0173	0.0050
Native American	222 (0)	246 (0)	0.0072	0.2441
Other race	1,705 (3)	1,861 (4)	0.0173	0.0050
Unknown race	8,933 (17)	10,698 (20)	0.0884	<0.0001^a^
Hispanic/Latino	4,391 (8)	3,425 (7)	0.0686	<0.0001^a^
Not Hispanic	34,408 (65)	37,319 (62)	0.0639	<0.0001^a^
Unknown ethnicity	14,118 (27)	16,612 (32)	0.1073	<0.0001^a^

*Note:* N = number of patients; Std Diff = standardised difference; SD = standard deviation.

a Significant at Bonferroni-corrected level of *p* = 0.0033. Entries in bold are of standardized differences above 0.2.

#### Developmental factors

A history of abuse in childhood or of family history of mental illness, or of conflict between the patient and parents, were all rarely coded, but all were more common in patients with FS than FMD, with negligible effects (see Table 2).

**Table 2.** Childhood or family factors coded in matched patients diagnosed with functional motor symptoms (FMDs) and functional seizures (FSs)

Characteristic	FMD, *n* (%)	FS, *n* (%)	Std Diff	*p*-value
History of any abuse in childhood	412 (1)	1,234 (4)	0.1266	<0.0001^a^
Family history of mental illness	1,015 (2)	1,414 (3)	0.0507	<0.0001^a^
Parent–child conflict	88 (0)	159 (1)	0.0280	<0.0001^a^
Problems related to upbringing	586 (1)	1,499 (3)	0.1252	<0.0001^a^

*Note:* N = number of patients; Std Diff = standardised difference.

a Significant at Bonferroni-corrected level of *p* = 0.012.

#### Baseline comorbidity

Almost all mental disorders, as well as migraine and a history of allergies, were more common in FS than in FMD, whereas somatoform disorders and functional somatic syndromes were more common in FMD. Depressive episodes had previously been diagnosed in 38% of people with FS, drug and alcohol problems in 26%, migraines in 23%, and bipolar disorder in 12%. All standardized differences were negligible except for drug and alcohol problems, post-traumatic stress disorder (PTSD), depressive episodes, and history of self-harm, which were more common in FS with small effects, and somatoform disorders and functional somatic syndromes, which were more common in FMD with small-medium effects (see Table 3).

**Table 3.** Previously recorded diagnoses at the time of diagnosis of functional motor symptoms (FMDs) and functional seizures (FSs)

Characteristic	FMD, *n* (%)	FS, *n* (%)	Std Diff	*p*-value
**Drug or alcohol problem**	**9,694 (19)**	**14,935 (29)**	**0.2379**	**<0.0001** ^a^
Schizophrenia	956 (2)	1,944 (4)	0.1155	<0.0001^a^
Bipolar disorder	3,433 (7)	6,377 (12)	0.1940	<0.0001^a^
**Depressive episode**	**14,592 (28)**	**19,887 (38)**	**0.2186**	**<0.0001** ^a^
Generalized anxiety disorder	5,404 (10)	7,949 (15)	0.1462	<0.0001^a^
Panic disorder	2,736 (5)	4,310 (8)	0.1203	<0.0001^a^
Depersonalization-derealization	24 (0)	32 (0)	0.0066	0.2849
**PTSD**	**4,016 (8)**	**7,849 (15)**	**0.2327**	**<0.0001** ^a^
Borderline personality disorder	1,024 (2)	1,974 (4)	0.1090	<0.0001^a^
Gender identity disorder	259 (0)	384 (1)	0.0306	<0.0001^a^
History of self-harm	818 (2)	2,529 (5)	0.1878	<0.0001^a^
Autistic disorder	584 (1)	1,124 (2)	0.0820	<0.0001^a^
Asperger’s syndrome	181 (0)	231 (0)	0.0153	0.0136
ADHD	3,123 (6)	4,508 (9)	0.1026	<0.0001^a^
**Somatoform disorder**	**7,138 (14)**	**1,915 (4)**	**0.3631**	**<0.0001** ^a^
Other headache syndrome	9,153 (18)	6,723 (13)	0.1306	<0.0001^a^
Migraine	8,835 (17)	12,501 (24)	0.1758	<0.0001^a^
Fibromyalgia	4,592 (9)	3,926 (8%)	0.0468	<0.0001^a^
**Post-viral fatigue syndrome**	**6,555 (13)**	**2,395 (5)**	**0.2890**	**<0.0001** ^a^

*Note:* ADHD = attention-deficit hyperactivity disorder; N = number of patients; PTSD = post-traumatic stress disorder; Std Diff = standardized difference.

a Significant at Bonferroni-corrected level of *p* = 0.0027. Entries in bold are of standardized differences above 0.2.

#### Antecedent conditions

**Figure 2.**
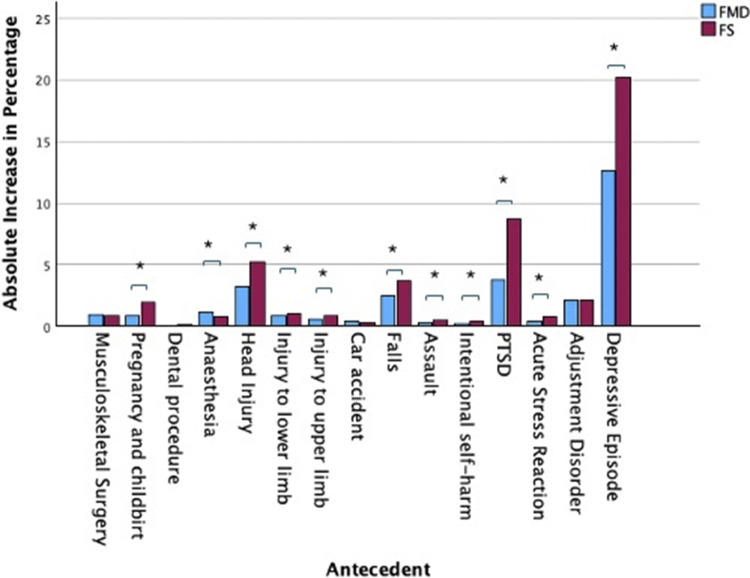
Absolute percentage increase in events in the 6 months before Functional Motor Symptoms (FMD) and Functional Seizures (FS) onset from a year earlier. PTSD = post-traumatic stress disorder. *Significant at Bonferroni corrected level of p=0.003.

Acute physical and mental health stressors in the 6 months preceding the onset of FMD and FS, and in the same period a year earlier, are presented in Supplementary Table 1. All stressors showed an increase in frequency in both diagnostic groups in the 6 months preceding onset compared with a year earlier (all increases statistically significant, except for dental procedures in both groups, see Supplementary Table 1), and most showed a differential association with FMD or FS that was the same across both time periods, though most events were rare, and most differences were negligible. The exceptions were PTSD and depressive episode, which showed much larger jumps from a year before to the 6 months preceding the onset of FS (from 2% to 11% for PTSD, and from 7% to 27% for depressive episode), with significantly smaller jumps before FMD (1–5% and 6–18%, respectively). The difference in the change in proportion between the groups was significant for all antecedents, except musculoskeletal surgery, dental procedures, car accidents, and adjustment disorder (Figure 2).

## Discussion

Our database included over 120,000 subjects, approximately evenly divided between FMD and FS. Their presentations confirmed all of the findings suggested by earlier studies, except that the effects of country of origin and dissociation could not be confirmed. People with FS were on average 8 years younger and diagnosed 6 years earlier than people with FMD. While there was no measure of country of origin available, there were significant differences in ethnicity, with Asian people being almost three times more likely to develop FMD than FS, and Black people slightly more likely to develop FS. Rates of childhood abuse were higher in FS, though rarely recorded, as were rates of anxiety, suicidal behavior, and borderline personality disorder. Indeed, most mental health conditions were significantly more common in FS, including, for example, depression, substance abuse, schizophrenia, and ADHD. Dissociation, when defined by depersonalization or derealization, showed no difference between groups, and was not found in significant rates in either. Chronic Fatigue Syndrome, fibromyalgia, nonmigraine headaches, and other somatoform disorders were more common in FMD, though migraines were more common in FS. In terms of what may be considered precipitants, there was an increase in injury and surgery in the 6 months before onset for both groups, compared with a year earlier, though surgery was more common preceding FMD than FS, and injury was more common in FS than FMD, and recorded rates for both groups were very low. Both stress reactions and depression doubled in the 6 months preceding onset in each group compared with a year earlier, and were higher in all cases in FS than FMD, suggesting psychological stressors were potentially common causes for both, but particularly FS.

These confirm the hypothesized differences between FMD and FS. They appear to occur in different people (notably younger people in FS); to be caused, or at least preceded, by different things; and to manifest different clinical associations, potentially supporting different mechanisms. FMDs are more likely to be preceded by surgery on their limbs and to be associated with somatoform disorders and functional somatic syndromes. FSs are more likely to be preceded by psychological injury – PTSD, depression, stress reactions – and to be associated with a wide range of mental illnesses. This leaves broad scope for interpretation, but one possibility would be a fundamental, if only partial, difference in their mechanisms of somatization and psychologization (Bridges, Goldberg, Evans, & Sharpe, 1991). Such differences have historically motivated diagnostic distinctions between depression and neurasthenia, for example, and would again be motivation for a diagnostic distinction between FMD and FS. There has long been a question over which ‘chapter’ FND belongs to, in answer to which the classification systems ICD and DSM differ. It could be that FMD and FS belong in different chapters, therefore, with FMD in the somatoform chapter, along with somatic symptom disorder, while FS perhaps belongs in those of stress reactions, along with PTSD.

The other key distinction is in etiology. The largest difference between the two groups was in age, and the most striking difference was the shape of the age distributions – FS was heavily skewed toward a post-puberty onset, whereas FMD appears to occur more evenly throughout adult life, peaking in middle age. When combined with the data on increased developmental risk factors and the preponderance of psychological antecedents in FS, these provide some support for the model we tentatively offered in our review (Kanaan et al., 2017): that FS may be considered in part a developmental disorder, where a more substantial risk burden leads to the early onset of symptoms when facilitated by the psychological and hormonal maelstrom of puberty and young adulthood, when young women in particular become vulnerable to common mental disorders (Kuehner, 2017). That FMD tends to occur throughout life suggests a different risk profile, perhaps that it depends on the co-occurrence of some other trigger which occurs later in life, such as a health, workplace, or marital event (Morsy et al., 2022).

We did not find the difference hypothesized in comorbid dissociation. Unfortunately, ICD-10 does not include any measure of the kinds of dissociation described in FND (Brown, 2006). We considered derealization-depersonalization, as perhaps the closest, but this is only coded when it occurs outside of another diagnosis: our hypothesized difference was in *ictal* dissociation or, more generally, dissociation occurring as part of the FND presentation, so probably cannot be tested with ICD-10 codes.

As the studies that led to our hypotheses were small, it may be surprising that the hypotheses were largely confirmed. As our sample was over 100 times larger than even the largest of those, it would have statistical power to find very small differences, but the size of the effects merits some discussion in tempering this. First, the absolute size of some of the effects appeared very small – implausibly so. To take one example, other large-scale studies suggest the lifetime risk of any mental disorder in the general population is ~50% (McGrath et al., 2023), so a history of mental illness in a family of any size should therefore be approaching 100%, yet our recorded family history was only 1–2%. There may be many reasons for underreporting or under-recording of family history, but the key one for this study is probably that the TrinetX database is, above all, an administrative record of clinical encounters. The items that will be recorded for each visit are likely to be things that are administratively important, such as billing, discharge diagnosis, the reason for the visit, and complicating factors. A history of mental illness in the patient’s family may well be recorded in their clinical file, but it is unlikely to make the final administrative summary, as it is unlikely to be of material difference. For this reason, many of the recorded factors – the childhood factors from Table 2, the adult factors from Table 4 – cannot be taken as fair estimates of their prevalence in these cohorts. The *differences* between these factors could be of undiminished value if the biases were the same toward both FMD and FS, but as the extent of the selection bias is clearly extreme, and the biases are unknown, they must be interpreted with great care. It is very possible that clinicians already aware of ideas about the frequency of childhood abuse in FS will be more likely to look for it, and code for it in FS, for example.

Yet the differences were, in most cases, small, too. One of the dangers of exploring a database of this size is that it may validate statistically significant differences that are too small to be of clinical significance. Panic disorder, for example, was found in 7% of FSs and only 5% of FMDs, a difference that was highly statistically significant, yet represents a standardized difference regarded as trivial (<0.2). This means that most people with FS and FMD will not differ in regard to their history of panic disorder, and at least when considered alone, this cannot represent a defining characteristic. Perhaps further significance may accrue to the difference, however, because it is argued to support an etiological or mechanistic feature of FS (Kanaan et al., 2022), and thus point to a difference which is of clinical significance. Indeed, each of the features considered in this article has been linked to hypothetical etiologies or mechanisms in FS or FMD.

The consideration of etiological factors does require particular care, however, as timing is a problem in three ways relevant to this study. The first is to note that, as above, these factors are probably only recorded during clinical encounters – they will not be backdated. Someone who presents to their doctor with a new onset of FMD and tells their doctor that it followed an injury a month ago is not going to have their FMD and injury recorded on separate dates, but will have them recorded on the same date (the day the information is coded). For this reason, we chose to include diagnoses and events recorded on *the same day* as the initial diagnosis of FND as part of the 6-month antecedents, though this will undoubtedly inflate the incidence of these factors – it would include, for example, a head injury that only occurred when a new onset of FS led to a fall. Second, the diagnosis of FND is fraught with delay in many cases, as tests are conducted or uncertainty remains, often for years (Jones et al., 2010). This should serve, however, to loosen the temporal link between antecedent and FND onset, so should have a conservative effect; but combined with the first problem, it means it is hard to say with confidence that the depression, for example, which appears to strongly prefigure the onset of FS, may not represent to some degree a consequence of it, rather than a cause. Third, our consideration of etiological factors was as much a comparison within groups (compared with a year earlier) as it was between groups, relying on an increase in the frequency of events preceding onset to indicate causal relevance. But such increases may occur for reasons that have nothing to do with etiology. It may be that depression, for example, becomes more likely after a year just as a function of age, rather than disease, and that this is differentially so in groups of different ages (more so in those teenagers with FS, for example, than those middle-aged with FMD – an additional control group would be needed to account for these effects.

Finally, we explicitly excluded those with both FMD and FS from our primary analyses. Our cohorts were defined by excluding anyone with a lifetime diagnosis of the other disorder, which represented just over 20% of each cohort. This group may be thought to have had the two diagnoses separately, since if they had them together they would arguably have been diagnosed “with mixed symptoms” (F44.7); however, we also did not include the group diagnosed with “mixed symptoms,” most of whom would probably have had FMD and FS together, as the most common FND symptoms, and represent a similar sized group again to those diagnosed with both separately. Combining these together would give an ‘overlap’ group a little less than half the size of either the FS or FMD groups. We excluded them to ensure the purity of our cohorts, but this served to maximize the differences between them, and of course, to conceal the overlap itself. The size of this overlap is itself a strong reason for wishing to retain FS and FMD in the same diagnostic category, even as the same disorder, but cannot by itself be enough to argue for diagnostic unity, since one of the findings of this paper (and others) is that both FS and FMD overlap with many other disorders, such as depression or pain, in very large proportions.

While there are many factors that go into deciding whether two conditions should be treated as different disorders (Wessely et al., 1999), these data at least support the idea that they are distinctive subtypes, with differences in symptoms, epidemiology, and etiology. And regardless of nosology, the differences we found here will add to the clinical picture of the two presentations. But despite the clear overlap in their presentations, their differences may point to potential differences in mechanism, which remains the ultimate ground for ‘splitting’ rather than ‘lumping’ together. Though psychiatric classification has been determined to be etiologically neutral since DSM-III, when etiological or mechanistic differences are found, they assume predominance, as in the case of PTSD. While everyone expects the etiology or mechanism of FMD or FS to be biopsychosocially complex, that does not mean they will be identical. The models enjoying greatest popularity for FMD (Edwards, Adams, Brown, Parees, & Friston, 2012) and FS (Brown & Reuber, 2016) do show overlap, but are also distinctly different. Until their mechanisms are confirmed, the question of whether to lump or split must remain a pragmatic one: whether this separation is useful to clinicians or researchers. But as the substantial separation of both research and clinical practice between FS and FMD was one of the facts motivating this study, that may be a question the field has already answered.

## Supporting information

10.1017/S0033291726103456.sm001 Kanaan et al. supplementary material Kanaan et al. supplementary material

## Data Availability

All data generated or analyzed during this study are included in this article and its Supplementary Material. Further enquiries can be directed to the corresponding author.

## Supplementary material

The supplementary material for this article can be found at http://doi.org/10.1017/S0033291726103456.
