# Supplementary material for: Evidence for a diagnostic distinction between functional seizures and functional motor symptoms from the TriNetX electronic health record database
Source: Psychol Med. 2026 Feb 26;56:e53. doi: 10.1017/S0033291726103456 (PMC12969201; doi:10.1017/S0033291726103456)
Supplement: Kanaan et al. supplementary material [file S0033291726103456sup001.docx]

| **Characteristic** | **0-6 months prior to FMD n (%)** | **12-18 months prior to FMD n (%)** | **Change in proportion p-value** | **0-6 months prior to FS n (%)** | **12-18 months prior to FS n (%)** | **Change in proportion p-value** | **Difference in proportions p-value** |
| --- | --- | --- | --- | --- | --- | --- | --- |
| Musculoskeletal Surgery | 1654 (3%) | 1286 (2%) | <0.0001* | 1199 (2%) | 844 (1%) | <0.0001* | 0.303 |
| Pregnancy and childbirth | 778 (1%) | 390 (1%) | <0.0001* | 1610 (3%) | 625 (1%) | <0.0001* | <0.0001* |
| Dental procedure | 108 (0%) | 89 (0%) | 0.3846 | 138 (0%) | 104 (0%) | 0.0648 | 0.0135 |
| Anaesthesia | 1380 (3%) | 858 (1%) | <0.0001* | 1067 (2%) | 734 (1%) | <0.0001* | <0.0001* |
| Head Injury | 2579 (5%) | 974 (2%) | <0.0001* | 4245(8%) | 1652 (3%) | <0.0001* | <0.0001* |
| Injury to lower limb | 1182 (2%) | 787 (1%) | <0.0001* | 1235 (2%) | 756 (1%) | <0.0001* | <0.0001* |
| Injury to upper limb | 792 (1%) | 519 (1%) | <0.0001* | 1028 (2%) | 665 (1%) | <0.0001* | <0.0001* |
| Car accident | 239 (0%) | 70 (0%) | <0.0001* | 248 (0%) | 121 (0%) | <0.0001* | 0.139 |
| Falls | 1963 (4%) | 705 (1%) | <0.0001* | 2979 (6%) | 1123 (2%) | <0.0001* | <0.0001* |
| Assault | 198 (0%) | 74 (0%) | <0.0001* | 401 (1%) | 194 (0%) | <0.0001* | <0.0001* |
| Intentional self-harm | 103 (0%) | 27 (0%) | <0.0001* | 276 (1%) | 88 (0%) | <0.0001* | <0.0001* |
| PTSD | 2622 (5%) | 696 (1%) | <0.0001* | 5908 (11%) | 1414 (2%) | <0.0001* | <0.0001* |
| Acute Stress Reaction | 276 (1%) | 91 (0%) | <0.0001* | 532 (1%) | 129 (0%) | <0.0001* | <0.0001* |
| Adjustment Disorder | 1492 (3%) | 424 (1%) | <0.0001* | 1503 (3%) | 427 (1%) | <0.0001* | 0.0051 |
| Depressive Episode | 9752 (18%) | 3309 (6%) | <0.0001* | 14559 (27%) | 4258 (7%) | <0.0001* | <0.0001* |

**Supplementary Table 1:** **Changes in** **antecedent Conditions recorded in the 6 months and the 12-18 months prior to the diagnosis of Functional Motor Symptoms (FMD) or Functional Seizures (FS).** N=number of patients; *Significant at Bonferroni corrected level of p=0.0017.
